# 1-(Phenyl­sulfon­yl)naphthalene

**DOI:** 10.1107/S1600536812030929

**Published:** 2012-07-14

**Authors:** Ying Fu, Wenbo Zhu, Hongxia Hou, Yinxia He, Hulin Li

**Affiliations:** a College of Chemistry and Chemical Engineering, Northwest Normal University, Lanzhou, An’ning East Road No. 967, Gansu Province 730070, People’s Republic of China

## Abstract

In the title compound, C_16_H_12_O_2_S, the phenyl ring is nearly perpendicular to the naphthalene system [dihedral angle = 80.3 (1)°]. The packing is consolidated by a weak C—H⋯π inter­action involving neighbouring naphthalene and benzene rings. In addition, there exist two different offset π–π stacking inter­actions between benzene rings and between naphthalene systems of symmetry-related mol­ecules [centroid–centroid distances = 3.876 (9) and 3.566 (4) Å, and slippage = 1.412 and 0.554 Å, respectively.

## Related literature

For recent reports on the synthesis of aryl­sulfones, see: Boroujeni (2010 ▶); Bahrami *et al.* (2008 ▶). For their application, see: Borys *et al.* (2012 ▶); Padwa *et al.* (1990 ▶); Block (1992 ▶); Mackinnon & Wang (1998 ▶). For single-crystal structures of sulfones, see: Chawdhury & Hargreaves (1971 ▶); Bacon & Curry (1960 ▶); Sime & Abrahams (1960 ▶); Jeyaraman & Velmurugan (1997 ▶).
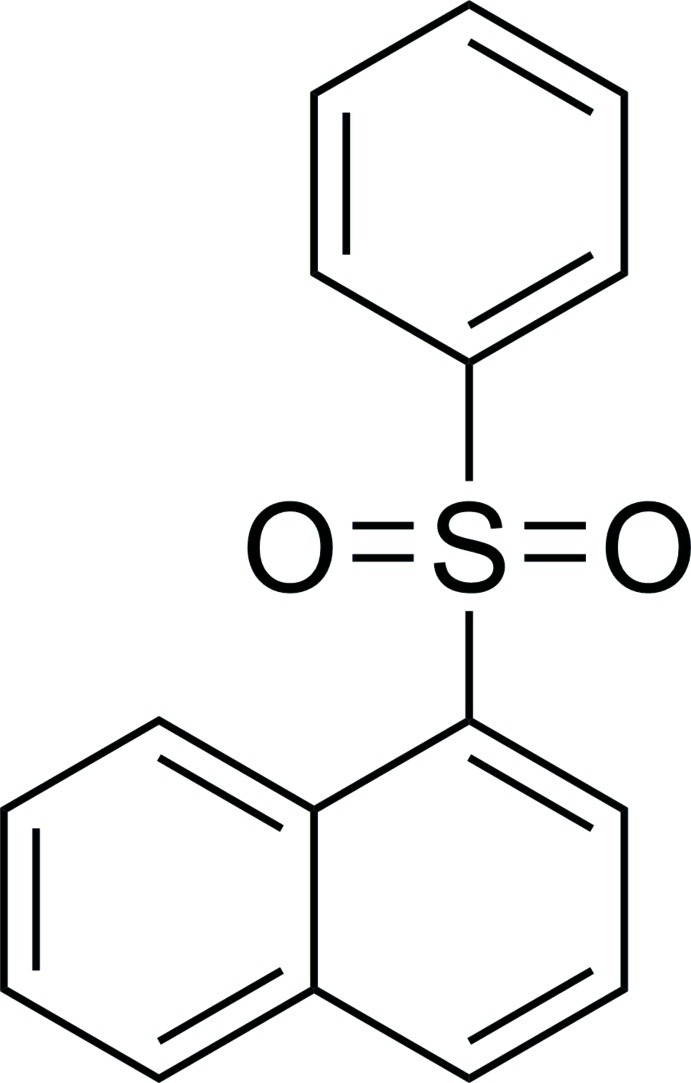


## Experimental

### 

#### Crystal data

C_16_H_12_O_2_S
*M* *_r_* = 268.32
Triclinic, 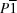

*a* = 7.721 (7) Å
*b* = 9.444 (9) Å
*c* = 9.726 (9) Å
α = 86.669 (8)°
β = 74.690 (8)°
γ = 69.995 (7)°
*V* = 642.4 (10) Å^3^
*Z* = 2
Mo *K*α radiation
μ = 0.25 mm^−1^
*T* = 296 K
0.25 × 0.23 × 0.19 mm

#### Data collection

Bruker APEXII CCD diffractometer
Absorption correction: multi-scan (*SADABS*; Bruker, 2004 ▶) *T*_min_ = 0.941, *T*_max_ = 0.955
4623 measured reflections
2345 independent reflections
1944 reflections with *I* > 2σ(*I*)
*R*_int_ = 0.021

#### Refinement

*R*[*F* ^2^ > 2σ(*F* ^2^)] = 0.038
*wR*(*F* ^2^) = 0.097
*S* = 1.05
2345 reflections
172 parameters
H-atom parameters constrained
Δρ_max_ = 0.17 e Å^−3^
Δρ_min_ = −0.32 e Å^−3^

### 

Data collection: *APEX2* (Bruker, 2004 ▶); cell refinement: *APEX2*; data reduction: *APEX2*; program(s) used to solve structure: *SHELXS97* (Sheldrick, 2008 ▶); program(s) used to refine structure: *SHELXL97* (Sheldrick, 2008 ▶); molecular graphics: *SHELXTL* (Sheldrick, 2008 ▶); software used to prepare material for publication: *SHELXTL*.

## Supplementary Material

Crystal structure: contains datablock(s) I, global. DOI: 10.1107/S1600536812030929/lr2072sup1.cif

Structure factors: contains datablock(s) I. DOI: 10.1107/S1600536812030929/lr2072Isup2.hkl

Supplementary material file. DOI: 10.1107/S1600536812030929/lr2072Isup3.mol

Supplementary material file. DOI: 10.1107/S1600536812030929/lr2072Isup4.cml

Additional supplementary materials:  crystallographic information; 3D view; checkCIF report

## Figures and Tables

**Table 1:** Hydrogen-bond geometry (Å, °) *Cg* is the centroid of the C11–C16 benzene ring.

*D*—H⋯*A*	*D*—H	H⋯*A*	*D*⋯*A*	*D*—H⋯*A*
C6—H6⋯*Cg* ^i^	0.93	2.90	3.806 (5)	166

Symmetry code: (i) 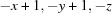.

**Fig. 1.**
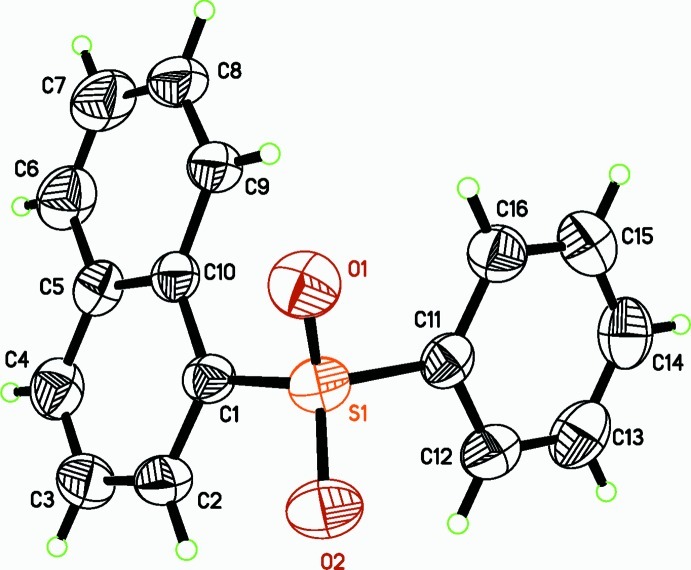
The molecular structure of (I), with atom labels and 50% probability displacement ellipsoids.

## supplementary crystallographic information

### Comment

Phenyl sulphone derivatives are an important class of organic sulfur compounds due to their broad spectrum of biological activities in a wide range of fields such as agrochemicals (Borys *et al.* 2012), pharmaceuticals (Padwa *et al.* 1990; Block, 1992) and polymers (Mackinnon & Wang, 1998). The molecular structure is shown in Fig. 1. All bond lengths and angles are in the normal range.

In the molecule of (I),Figure 1, both the naphthalene ring and the phenyl ring adopt essentially planar conformation with a maximum deviation of 0.0032 Å for the phenyl ring and 0.0038 Å for the naphthalene ring. The phenyl ring is nearly perpendicular to the naphthalene ring with the dihedral angle 80.3 (1)°.

The packing is stabilized by a weak C6–H6···π interaction, involving neighbouring naphthalene and benzene rings (*-x+1,-y+1,-z)* . In addition, there exist two different offset π—π stacking interactions one between the benzene and another between the naphthalene rings of symmetry-related molecules (*via -x+1, -y, -z+1* and *-x,-y+1, -z* operations respectively), with centroid-centroid distances= 3.876 (9) and 3.566 (4), slippage = 1.412 and 0.554 Å respectively.

### Experimental

For the synthesis of the title compound,see: Boroujeni (2010); Crystals suitable for single-crystal X-ray analysis were grown by slow evaporation of a solution in petroleum ether-ethyl acetate (5:1).

### Refinement

All H atoms were geometrically positioned and refined using a riding model with C—H = 0.93 Å, *U*_iso_(H) = 1.2 *U*_eq_(C).

### Crystal data

**Table:**

C_16_H_12_O_2_S	*Z* = 2
*M**_r_* = 268.32	*F*(000) = 280
Triclinic, *P*1	*D*_x_ = 1.387 Mg m^−^^3^
Hall symbol: -P 1	Mo *K*α radiation, λ = 0.71073 Å
*a* = 7.721 (7) Å	Cell parameters from 2200 reflections
*b* = 9.444 (9) Å	θ = 2.2–27.4°
*c* = 9.726 (9) Å	µ = 0.25 mm^−^^1^
α = 86.669 (8)°	*T* = 296 K
β = 74.690 (8)°	Block, colourless
γ = 69.995 (7)°	0.25 × 0.23 × 0.19 mm
*V* = 642.4 (10) Å^3^	

### Data collection

**Table:**

Bruker APEXII CCD diffractometer	2345 independent reflections
Radiation source: fine-focus sealed tube	1944 reflections with *I* > 2σ(*I*)
Graphite monochromator	*R*_int_ = 0.021
φ and ω scans	θ_max_ = 25.5°, θ_min_ = 2.2°
Absorption correction: multi-scan (*SADABS*; Bruker, 2004)	*h* = −9→8
*T*_min_ = 0.941, *T*_max_ = 0.955	*k* = −11→10
4623 measured reflections	*l* = −11→11

### Refinement

**Table:**

Refinement on *F*^2^	Primary atom site location: structure-invariant direct methods
Least-squares matrix: full	Secondary atom site location: difference Fourier map
*R*[*F*^2^ > 2σ(*F*^2^)] = 0.038	Hydrogen site location: inferred from neighbouring sites
*wR*(*F*^2^) = 0.097	H-atom parameters constrained
*S* = 1.05	*w* = 1/[σ^2^(*F*_o_^2^) + (0.0457*P*)^2^ + 0.1481*P*] where *P* = (*F*_o_^2^ + 2*F*_c_^2^)/3
2345 reflections	(Δ/σ)_max_ < 0.001
172 parameters	Δρ_max_ = 0.17 e Å^−^^3^
0 restraints	Δρ_min_ = −0.32 e Å^−^^3^

### Special details

**Table:**

Geometry. All e.s.d.'s (except the e.s.d. in the dihedral angle between two l.s. planes) are estimated using the full covariance matrix. The cell e.s.d.'s are taken into account individually in the estimation of e.s.d.'s in distances, angles and torsion angles; correlations between e.s.d.'s in cell parameters are only used when they are defined by crystal symmetry. An approximate (isotropic) treatment of cell e.s.d.'s is used for estimating e.s.d.'s involving l.s. planes.
Refinement. Refinement of *F*^2^ against ALL reflections. The weighted *R*-factor *wR* and goodness of fit *S* are based on *F*^2^, conventional *R*-factors *R* are based on *F*, with *F* set to zero for negative *F*^2^. The threshold expression of *F*^2^ > σ(*F*^2^) is used only for calculating *R*-factors(gt) *etc*. and is not relevant to the choice of reflections for refinement. *R*-factors based on *F*^2^ are statistically about twice as large as those based on *F*, and *R*- factors based on ALL data will be even larger.

### Fractional atomic coordinates and isotropic or equivalent isotropic displacement parameters (Å^2^)

**Table:**

	*x*	*y*	*z*	*U*_iso_*/*U*_eq_	
C1	0.2011 (2)	0.34105 (19)	0.13095 (18)	0.0371 (4)	
C2	0.2381 (3)	0.2563 (2)	0.0103 (2)	0.0471 (5)	
H2	0.2356	0.1584	0.0184	0.056*	
C3	0.2797 (3)	0.3150 (2)	−0.1249 (2)	0.0552 (5)	
H3	0.3031	0.2566	−0.2059	0.066*	
C4	0.2859 (3)	0.4559 (2)	−0.1378 (2)	0.0542 (5)	
H4	0.3140	0.4938	−0.2282	0.065*	
C5	0.2506 (3)	0.5470 (2)	−0.0172 (2)	0.0448 (5)	
C6	0.2557 (3)	0.6953 (3)	−0.0325 (3)	0.0622 (6)	
H6	0.2860	0.7320	−0.1233	0.075*	
C7	0.2173 (4)	0.7842 (3)	0.0828 (3)	0.0703 (7)	
H7	0.2213	0.8815	0.0712	0.084*	
C8	0.1712 (3)	0.7305 (2)	0.2201 (3)	0.0583 (6)	
H8	0.1450	0.7929	0.2989	0.070*	
C9	0.1642 (3)	0.5888 (2)	0.2401 (2)	0.0460 (5)	
H9	0.1324	0.5557	0.3322	0.055*	
C10	0.2049 (2)	0.49129 (19)	0.12201 (19)	0.0373 (4)	
C11	0.3619 (3)	0.21084 (19)	0.35340 (18)	0.0392 (4)	
C12	0.5126 (3)	0.0821 (2)	0.2971 (2)	0.0502 (5)	
H12	0.5019	0.0197	0.2316	0.060*	
C13	0.6790 (3)	0.0470 (3)	0.3391 (3)	0.0609 (6)	
H13	0.7810	−0.0398	0.3021	0.073*	
C14	0.6953 (3)	0.1394 (3)	0.4350 (2)	0.0579 (6)	
H14	0.8086	0.1158	0.4619	0.070*	
C15	0.5448 (3)	0.2663 (3)	0.4913 (2)	0.0577 (5)	
H15	0.5562	0.3282	0.5569	0.069*	
C16	0.3766 (3)	0.3030 (2)	0.4514 (2)	0.0494 (5)	
H16	0.2743	0.3889	0.4901	0.059*	
O1	−0.00281 (18)	0.35703 (15)	0.39697 (14)	0.0523 (4)	
O2	0.1356 (2)	0.11219 (15)	0.26565 (15)	0.0560 (4)	
S1	0.15229 (6)	0.25301 (5)	0.29564 (5)	0.04155 (17)	

### Atomic displacement parameters (Å^2^)

**Table:**

	*U*^11^	*U*^22^	*U*^33^	*U*^12^	*U*^13^	*U*^23^
C1	0.0317 (9)	0.0348 (9)	0.0422 (9)	−0.0083 (7)	−0.0084 (8)	−0.0034 (7)
C2	0.0484 (11)	0.0388 (10)	0.0508 (11)	−0.0095 (9)	−0.0128 (9)	−0.0070 (8)
C3	0.0608 (14)	0.0552 (13)	0.0421 (11)	−0.0088 (11)	−0.0126 (10)	−0.0109 (9)
C4	0.0538 (13)	0.0589 (13)	0.0405 (10)	−0.0100 (10)	−0.0093 (9)	0.0048 (9)
C5	0.0397 (10)	0.0430 (11)	0.0499 (11)	−0.0113 (8)	−0.0131 (9)	0.0061 (8)
C6	0.0703 (15)	0.0524 (13)	0.0690 (14)	−0.0276 (12)	−0.0209 (12)	0.0187 (11)
C7	0.0824 (17)	0.0420 (12)	0.0986 (19)	−0.0296 (12)	−0.0348 (15)	0.0123 (13)
C8	0.0635 (14)	0.0398 (11)	0.0764 (15)	−0.0139 (10)	−0.0290 (12)	−0.0083 (10)
C9	0.0474 (11)	0.0402 (11)	0.0519 (11)	−0.0119 (9)	−0.0180 (9)	−0.0044 (8)
C10	0.0301 (9)	0.0369 (10)	0.0445 (10)	−0.0088 (7)	−0.0120 (8)	−0.0007 (8)
C11	0.0402 (10)	0.0362 (10)	0.0388 (9)	−0.0140 (8)	−0.0061 (8)	0.0073 (8)
C12	0.0511 (12)	0.0403 (11)	0.0538 (12)	−0.0113 (9)	−0.0102 (10)	−0.0001 (9)
C13	0.0443 (12)	0.0537 (13)	0.0706 (14)	−0.0041 (10)	−0.0094 (11)	0.0084 (11)
C14	0.0475 (12)	0.0718 (15)	0.0585 (13)	−0.0240 (11)	−0.0187 (10)	0.0170 (11)
C15	0.0602 (14)	0.0688 (15)	0.0506 (12)	−0.0265 (12)	−0.0191 (11)	0.0026 (10)
C16	0.0504 (12)	0.0475 (11)	0.0454 (11)	−0.0125 (9)	−0.0093 (9)	−0.0014 (9)
O1	0.0391 (8)	0.0590 (9)	0.0487 (8)	−0.0127 (6)	0.0016 (6)	−0.0058 (6)
O2	0.0611 (9)	0.0454 (8)	0.0689 (9)	−0.0308 (7)	−0.0122 (7)	0.0019 (7)
S1	0.0389 (3)	0.0396 (3)	0.0451 (3)	−0.0161 (2)	−0.0051 (2)	0.00010 (19)

### Geometric parameters (Å, º)

**Table:**

C1—C2	1.369 (3)	C9—C10	1.415 (3)
C1—C10	1.426 (3)	C9—H9	0.9300
C1—S1	1.772 (2)	C11—C16	1.378 (3)
C2—C3	1.397 (3)	C11—C12	1.380 (3)
C2—H2	0.9300	C11—S1	1.763 (2)
C3—C4	1.345 (3)	C12—C13	1.377 (3)
C3—H3	0.9300	C12—H12	0.9300
C4—C5	1.407 (3)	C13—C14	1.370 (3)
C4—H4	0.9300	C13—H13	0.9300
C5—C6	1.413 (3)	C14—C15	1.369 (3)
C5—C10	1.423 (3)	C14—H14	0.9300
C6—C7	1.347 (3)	C15—C16	1.378 (3)
C6—H6	0.9300	C15—H15	0.9300
C7—C8	1.400 (3)	C16—H16	0.9300
C7—H7	0.9300	O1—S1	1.4335 (15)
C8—C9	1.358 (3)	O2—S1	1.4328 (18)
C8—H8	0.9300		
			
C2—C1—C10	120.89 (17)	C9—C10—C5	117.97 (18)
C2—C1—S1	116.45 (16)	C9—C10—C1	125.12 (17)
C10—C1—S1	122.66 (13)	C5—C10—C1	116.90 (16)
C1—C2—C3	120.9 (2)	C16—C11—C12	120.55 (19)
C1—C2—H2	119.6	C16—C11—S1	121.51 (15)
C3—C2—H2	119.6	C12—C11—S1	117.94 (15)
C4—C3—C2	120.03 (19)	C13—C12—C11	119.3 (2)
C4—C3—H3	120.0	C13—C12—H12	120.4
C2—C3—H3	120.0	C11—C12—H12	120.4
C3—C4—C5	121.27 (19)	C14—C13—C12	120.4 (2)
C3—C4—H4	119.4	C14—C13—H13	119.8
C5—C4—H4	119.4	C12—C13—H13	119.8
C4—C5—C6	120.6 (2)	C15—C14—C13	120.1 (2)
C4—C5—C10	120.02 (19)	C15—C14—H14	120.0
C6—C5—C10	119.37 (19)	C13—C14—H14	120.0
C7—C6—C5	120.8 (2)	C14—C15—C16	120.5 (2)
C7—C6—H6	119.6	C14—C15—H15	119.8
C5—C6—H6	119.6	C16—C15—H15	119.8
C6—C7—C8	120.2 (2)	C11—C16—C15	119.24 (19)
C6—C7—H7	119.9	C11—C16—H16	120.4
C8—C7—H7	119.9	C15—C16—H16	120.4
C9—C8—C7	121.1 (2)	O2—S1—O1	118.25 (10)
C9—C8—H8	119.4	O2—S1—C11	107.09 (9)
C7—C8—H8	119.4	O1—S1—C11	108.84 (10)
C8—C9—C10	120.5 (2)	O2—S1—C1	107.42 (10)
C8—C9—H9	119.7	O1—S1—C1	109.94 (10)
C10—C9—H9	119.7	C11—S1—C1	104.41 (9)
			
C10—C1—C2—C3	−0.4 (3)	C16—C11—C12—C13	−0.5 (3)
S1—C1—C2—C3	−179.51 (15)	S1—C11—C12—C13	179.31 (15)
C1—C2—C3—C4	0.8 (3)	C11—C12—C13—C14	−0.4 (3)
C2—C3—C4—C5	−0.2 (3)	C12—C13—C14—C15	0.9 (3)
C3—C4—C5—C6	−179.36 (19)	C13—C14—C15—C16	−0.5 (3)
C3—C4—C5—C10	−0.8 (3)	C12—C11—C16—C15	0.9 (3)
C4—C5—C6—C7	178.3 (2)	S1—C11—C16—C15	−178.94 (14)
C10—C5—C6—C7	−0.2 (3)	C14—C15—C16—C11	−0.4 (3)
C5—C6—C7—C8	−0.1 (4)	C16—C11—S1—O2	−148.12 (15)
C6—C7—C8—C9	0.0 (4)	C12—C11—S1—O2	32.06 (17)
C7—C8—C9—C10	0.5 (3)	C16—C11—S1—O1	−19.20 (18)
C8—C9—C10—C5	−0.8 (3)	C12—C11—S1—O1	160.98 (14)
C8—C9—C10—C1	−179.78 (18)	C16—C11—S1—C1	98.16 (17)
C4—C5—C10—C9	−177.90 (17)	C12—C11—S1—C1	−81.66 (17)
C6—C5—C10—C9	0.7 (3)	C2—C1—S1—O2	−8.25 (17)
C4—C5—C10—C1	1.2 (3)	C10—C1—S1—O2	172.61 (14)
C6—C5—C10—C1	179.75 (17)	C2—C1—S1—O1	−138.16 (15)
C2—C1—C10—C9	178.39 (18)	C10—C1—S1—O1	42.70 (17)
S1—C1—C10—C9	−2.5 (2)	C2—C1—S1—C11	105.24 (15)
C2—C1—C10—C5	−0.6 (3)	C10—C1—S1—C11	−73.90 (15)
S1—C1—C10—C5	178.48 (13)		

### Hydrogen-bond geometry (Å, º)

Cg is the centroid of the C11–C16 benzene ring.

**Table:**

*D*—H···*A*	*D*—H	H···*A*	*D*···*A*	*D*—H···*A*
C6—H6···*Cg*^i^	0.93	2.90	3.806 (5)	166

Symmetry code: (i) −*x*+1, −*y*+1, −*z*.
